# Editorial: Genetic and epigenetic regulatory mechanisms in higher plants in response to abiotic stress

**DOI:** 10.3389/fpls.2024.1374289

**Published:** 2024-02-02

**Authors:** Jemaa Essemine, Mokhtar Guerfel, Mingnan Qu

**Affiliations:** ^1^ National Key Laboratory of Plant Molecular Genetics, Chinese Academy of Science (CAS) Center for Excellence in Molecular Plant Sciences, Chinese Academy of Sciences, Shanghai, China; ^2^ Department of Biological Sciences, College of Education Afif, Shaqra University, Ar Riyadh, Saudi Arabia

**Keywords:** stress resilience, molecular biology, genetic, crop breeding, epigenetic, omics, NGS, CRISPR

Genetic and epigenetics regulatory biomarkers play an utmost importance in molecular mechanisms of plant tolerance to stress and crop breeding approaches. As adverse growth conditions hamper crop yields and global food security, feeding an ever-growing worldwide population represents an ever-challenging task. To well disentangle the aforementioned mechanisms, scientists have resorted to integrating several plant research fields, and so, they have to be well equipped with robust bioinformatics knowledge and tools to manage big datasets. Essentially, the regular articles encompassed in this topic deal with modern concerns facing farmers and stockholders. To solve these, scientists have applied a multifaceted research approach spanning various realms such as plant physiology, molecular biology, genetics, epigenetics, and omics in state-of-the-art plant science and cutting-edge approaches empowered by sophisticated technologies and advanced methodologies, including genome-wide association studies (GWAS) and epigenetic methods, to unravel the mechanism orchestrating plant tolerance to stresses (biotic and abiotic) such as heat, salinity, drought, and pathogen attacks, etc. Hence, evolved molecular techniques can be invested in future crop breeding strategies to enhance productivity and generate new varieties that are more resilient to environmental challenges and resistant to pathogen aggressions. Notably, Kumar et al. unveiled the crucial importance of the molecular basis of genetic plasticity to varying environmental conditions on growing rice using two different methods.

This topic gathers new findings and useful methodologies to foster plant science research. It sheds light on the role of epigenetics changes, such as DNA methylation, histone (de)acetylation, and other post-translational modifications (PTMs), in genes regulations (repression or induction) and that of omics (genomics, epigenomics, transcriptomics, metabolomics, ionomics, and proteomics) in detecting stress-responsive genes. Using inducible Clustered Interspaced Short Palindromic Repeat interference (iCRISPRi), Yapa et al. were able to reduce the *MORF2* transcripts in Arabidopsis seedlings. Thus, they revealed that reducing MORF2 by iCRISPRi stimulated the expression of stress-responsive genes, triggered plastidial retrograde signaling, repressed ethylene signaling and skotomorphogenesis, and increased accumulation of hydrogen peroxide (H_2_O_2_).

Moreover, Run et al. displayed in their study the eminent importance of wheat potassium transporter TaHAK_13_ in mediating K^+^ absorption and maintenance of potassium homeostasis under low potassium stress. Further, they proved, for *TaHAK_13_* gene cloned from wheat, using a qRT-PCR analysis, that *TaHAK13* expression can be induced by environmental stress and upregulated under drought, low potassium, and salt (NaCl). In the same trend, using the GUS staining technique, Run et al. indicated that *TaHAK13* was mainly expressed in leaf veins, stems, and root tips of Arabidopsis seedlings, and its expression varied with the developmental stage. Similarly, Fang et al. exhibited that overexpression of *ScRIPK* of sugarcane, a member of receptor-like cytoplasmic kinases (RLCKs; subfamily RLCK VII), in Arabidopsis enhanced drought tolerance and disease susceptibility.

Furthermore, this topic reported that exploiting recent advancements in data science and bioinformatic tools, including computational biology (dry experiment), has led to a striking leap in biotechnology analysis methods, as reported by Yapa et al. regarding iCRISPRi as a new technique. Thus, it is of great interest to apply multidisciplinary research activity fostered by new approaches to delimit plant stress resilience features and define suitable breeding strategies for improved crop productivity. Hence, genomics dissection helps to recognize stress-responsive genes, unravels regulatory networks, and characterizes genetic variations associated with stress-tolerance events. Contributors to this topic stressed that using transcriptomics profiling decodes the complex dynamics of gene expression during stress conditions, unearthing novel stress-responsive genes and signaling pathways.

The acquired competencies and compiled insights shape the advent and development of stress-tolerant crop varieties, achieved through conventional breeding programs and state-of-the-art techniques encompassing genetic engineering and gene-editing tools such as CRISPR-Cas9. Thus, the integration of diverse omics data and functional genomics approaches empowers precise manipulations of crop genomes to fortify their stress resilience. Incorporation of genomics and transcriptomics bears a substantial burden in elucidating the molecular mechanisms standing behind crop stress tolerance and may pave the way toward sustainable agriculture to preserve food security amidst shifting environmental challenges.

Based on genomic and omics methods, the contributors sought to dissect plant genome and determine potential candidate genes associated with physiological or agronomic traits using high-throughput technologies such as next-generation sequencing (NGS) and GWAS using CRISPR-Cas9-based genome-editing system. This helps to efficiently identify crucial biomarkers (gene(s) or TFs) monitoring crop yield-related traits including grain size and grain weight, etc. Through editing the concerned genes (knock-out, Indel, etc.) accompanied by some validating wet experiments (qRT-PCR, transformations) and omics analysis and profiling, scientists and farmers may successfully improve plant yield via effective breeding programs. Breeders seek to ameliorate plant plasticity to withstand severe stress conditions and, thereby, their resilience and productivity.

The organized topic was further dedicated to comprehending the molecular mechanism of plant cell memory of stress (abiotic and biotic) during next generation. This implies that the epigenetic regulation of stress memory by plant cell tissues and organs (root, leaf, flower) during next generation of culture may occur through DNA methylation, histone modifications, and/or other regulator modules (suppressor or inducer) including KRAB-HA and MORF2-2 (Yapa et al.). One of the possible yet unexplored ways to boost stress tolerance in crop plants might be a promoted plant memory through well-targeted epigenome modification.

Altogether, the content of this topic highlights the integration of various research domains, including plant molecular biology, genetics, epigenetic regulatory factors, omics, and synthetic biology (Synbio) in plants. To pinpoint the intricate and interconnected cellular processes in the plant kingdom, researchers have adopted integrative omics and computational analyses spanning various biological systems. The combination of various omics data might unbiasedly help explain the interplay between different biological processes and pathways to dissect their role in plant development, resistance to stress, and cross-breeding likelihood.

Overall, well deciphering the molecular, genetic, and epigenetic regulatory mechanisms of plant resilience to stress may open new perspectives and illustrate purposeful breeding strategies to improve crop production and create new varieties more resilient to global warming, climate changes, and/or pathogens invasion. Thus, a substantial progress in gene-editing technologies has been made that efficiently facilitates the use of genetic variation in plants, thus paving new avenues toward revolutionized agriculture thanks to next-generation breeding approaches for the sake of continuous cropping and sustainable development.

## Author contributions

JE: Conceptualization, Investigation, Writing – original draft, Writing – review & editing. MG: Writing – review & editing. MQ: Writing – review & editing.

